# Evaluating large language models for automated REDCap support ticket triage and response

**DOI:** 10.1093/jamiaopen/ooag073

**Published:** 2026-06-05

**Authors:** Andrew Carroll, Dawn Brown, Domenick Silvio, James Maszatics, David A Hanauer

**Affiliations:** Michigan Institute for Clinical and Health Research, University of Michigan Medical School, Ann Arbor, MI, 48109, United States; Michigan Institute for Clinical and Health Research, University of Michigan Medical School, Ann Arbor, MI, 48109, United States; Michigan Institute for Clinical and Health Research, University of Michigan Medical School, Ann Arbor, MI, 48109, United States; Michigan Institute for Clinical and Health Research, University of Michigan Medical School, Ann Arbor, MI, 48109, United States; Michigan Institute for Clinical and Health Research, University of Michigan Medical School, Ann Arbor, MI, 48109, United States; Department of Learning Health Sciences, University of Michigan Medical School, Ann Arbor, MI, 48109, United States

**Keywords:** artificial intelligence, health information systems, large language models, natural language processing, office automation

## Abstract

**Objectives:**

To evaluate the utility of large language models (LLMs) without and with retrieval-augmented generation (RAG) resources for automating Research Electronic Data Capture (REDCap) support at a large academic medical center, focusing on ticket complexity, categorization, and response drafting.

**Materials and Methods:**

We randomly selected 90 REDCap support tickets from 6316 received in 2023, stratified by estimated complexity (low, moderate, high) using ticket metadata. Four LLM configurations (GPT-4o Default; GPT-4o + Vanderbilt REDCap documentation; GPT-4o + University of Michigan [UM] REDCap documentation; GPT-4o + combined documentation) were evaluated on 3 tasks per ticket: complexity assessment, category assignment, and email response drafting. Human REDCap experts provided gold-standard ratings and assessed email drafts.

**Results:**

Compared to the gold standard, the GPT-4o Default environment most closely matched human complexity ratings, while specialized RAG environments tended to overestimate complexity. For ticket support categorization, all LLM configurations achieved high agreement with human judgments (Cohen’s kappa ≈0.85), correctly categorizing 81%-89% of tickets. In generating ticket responses, the GPT-4o + REDCap + UM environment provided the most correct answers, particularly for low and moderate complexity tickets, where about two-thirds of responses were substantially or fully correct.

**Discussion:**

Large language models have potential to automate ticket triage and response drafting for routine REDCap support, with context augmentation improving response accuracy for lower complexity tickets.

**Conclusion:**

Large language model-based support tools hold promise for reducing human workload for REDCap support tickets. Continued human oversight, iterative prompt engineering, and expanded local documentation are needed for reliable, effective deployment.

## Introduction

With Research Electronic Data Capture (REDCap) in use at nearly 8000 institutions worldwide, its role in research data collection and management is unparalleled.^1^ The University of Michigan (UM) has hosted a REDCap instance supported by the Michigan Institute for Clinical and Health Research (MICHR) since July 2010. As of July 2025, we had 3692 active users and 9064 active projects in the system. In addition to technical support (eg, installing updates, monitoring servers), 3 MICHR staff handle all REDCap-related help tickets. The team estimates about 6000 h yearly are required to address and respond to all the REDCap tickets, or approximately 3 full time equivalents. This is roughly in line with the REDCap support requirements reported elsewhere.^2^ Given the limited research support resources, delays in ticket resolution can prevent study teams from collecting data and progressing with their research.

Advances in artificial intelligence (AI) and large language models (LLMs) are showing great promise for supporting customer service tasks in the general consumer space, where use of these technologies has increased problem resolution while simultaneously decreasing both response times and service costs.^3–5^ Based on current trends, these technologies are expected to become ubiquitous for customer support in just a few years.^6^^,^^7^

The use of LLMs for responding to customers (ie, patients) in the health-care delivery space is also gaining traction, especially for generating responses to patient portal messages.^8–10^ However, there remains a paucity of research on the use of LLMs in the health-care research space, which also requires responding to customers (ie, researchers).

Clinical and translational science efforts aim to increase research effectiveness and efficiency. Streamlining the ability to respond to the large volume of REDCap support tickets aligns with our mission to support and accelerate scientific discovery. In the interest of identifying novel approaches to more effectively address REDCap tickets, we sought to determine if AI-based LLMs had the potential to increase support staff productivity while maintaining reliability and accuracy. This study explored the performance of a widely available, well-regarded, commercial LLM with respect to handling REDCap-related support tickets.

## Materials and methods

In 2023, the MICHR team received 6316 support tickets related to REDCap, and it is these tickets that formed the basis of this study.

### Estimated ticket complexity

An initial task for this work was to select tickets of varying complexity for testing. Tickets are tracked in a commercial system called ServiceNow, which supports business processes including ticket management and progress. Some issues submitted through tickets are low complexity and quick to solve (eg, requests for simple new project approvals or enabling basic settings), whereas others are high complexity and more nuanced, unique, and time consuming (eg, issues with Automated Survey Invitations employing complex logic or multifaceted “how to” questions). However, our team does not typically label or track ticket complexity. The following analysis and scoring process was used solely for the purpose of stratifying tickets to select the sample for analysis; these scores were not used in the main analysis, results, or conclusions of the study.

We therefore developed an approach to estimate the complexity of tickets for our analyses. For all tickets tracked in ServiceNow, there are 2 metrics that we considered as proxies for the complexity of the issue submitted by users:

1. Number of updates. An update represents any time one of the MICHR staff made a change to the ticket as they worked through the issues. The number of updates here is used as a proxy for complexity, but it may be better understood as a proxy for the effort required to resolve a ticket, as there are likely situations where high-complexity tickets could have few updates, and less complex tickets have many updates. Despite being an imperfect proxy, our team believed that, in general, the more times a ticket needed to be updated, the more complex the problem was. The number of ticket updates for this dataset ranged from 2 to 55, with a mean of 6.7 updates per ticket (median=6 updates). Based on the update counts, the tickets were assigned a score of 1-3. Tickets with 5 or fewer updates were assigned a score of 1 (*n*=2000), those with 6 updates were assigned a score of 2 (*n*=1743), and those with 7 or more updates were assigned a score of 3 (*n*=2573). This grouping was based on practical considerations; we used the median value of 6 updates as its own group to split the ticket population into approximately 3 equal portions. There was no strong theoretical reason to designate 6 as its own group beyond achieving balanced group sizes. Although alternative groupings (such as defining a range for the moderate group) could have been used, we felt a simple data-driven approach was justified given the lack of established guidance for stratifying ticket complexity from update counts.
2. Time to resolution. In general, the longer a ticket remained open, the more difficult it was to solve the issue. (There are scenarios in which a ticket might remain open for longer than expected, such as when a ticket was opened just before a long holiday weekend and it might not have been addressed until the weekend was over.) This highlights circumstances that can affect time to resolution beyond ticket complexity; it is worth nothing that similar circumstances may also apply to number of updates, for example, a complex ticket may have few updates if an issue is identified and resolved in one communication, or a less complex issue could have many updates due to user inexperience. The total resolution times for the tickets ranged from 22 s to 9 057 307 s (104 days) with a mean of 64 091 s (17.8 h) and a median time of 3372 s (56.2 min). Based on the open ticket times, the tickets were assigned a score of 1-4: 1579 tickets were assigned a score of 1 (time range 22-760 s), 1579 tickets were assigned a score of 2 (time range 761-3371 s), 1579 tickets were assigned a score of 3 (time range 3372-37393 s), and 1579 tickets were assigned a score of 4 (time range 37 394-9 057 000 s). The use of 4 equal groups for time to resolution was chosen for even stratification across the sample size. The number of scores for updates (*n*=3) and time to resolution (*n*=4) differs, which was a decision based on the distributions within each metric.

Using the sum of the scores from ticket updates and the resolution times (ranging from 2 to 7), the tickets were divided again into 3 groups and assigned an estimated complexity score of low (total score 2-3), moderate (score 4-5), or high (score 6-7). Once these tickets were separated into 3 complexity groups (Table 1), we randomly sampled 30 tickets from each group to use in this study. The stratification groups (low, moderate, high) are based on the sum of the update and time scores, not to be confused with the separate outcome variable “ticket category” used later in the study.

**Table 1:** Number of tickets for each complexity score and grouped into low, moderate, and high complexity.

Ticket complexity group	Total score	Number of tickets per total score	Number of tickets per estimated ticket complexity group
Low	2	749	1716
	3	967	
Moderate	4	1279	2597
	5	1318	
High	6	1131	2003
	7	872	

Thirty tickets from each ticket complexity group were randomly selected for use in our experiments.

The rationale for collapsing the 6 possible scores to 3 final groups of low, moderate, and high complexity was to provide groups of sufficient size for random sampling and analysis. The decision to sample 30 tickets per group was based on practical considerations, but we acknowledge that distributing sampling across more possible score groups would have been an alternative approach.

### Large language model infrastructure

The UM supports multiple secure, privacy-protected LLM integrations, including options from vendors such as OpenAI (the creator of ChatGPT) and Anthropic. One notable offering is the “Maizey” service, which enables members of the UM community to create chatbots powered by LLMs that are enhanced with user-uploaded data resources. Users can upload their own datasets—such as documents, spreadsheets, or policy manuals—which Maizey processes by indexing content with vector-embeddings. When users submit queries, Maizey retrieves relevant document sections using semantic search and supplies this information to the LLM to generate accurate, context-specific answers, a process known as retrieval-augmented generation (RAG). Maizey has been employed across the university to create course or departmental knowledge assistants and to support administrative processes, allowing units to upload materials and enable Maizey to assist end users with tailored information retrieval. Notably, uploaded documents are not used to train the LLM but rather to inform responses generated by the LLM during each session.

For these experiments, we utilized 4 LLM environments:

1. *GPT-4o Default*: This was the standard GPT-4o model provided by OpenAI at the time of the experiments, with no additional data added for answering questions. However, we presume that some REDCap documentation would have been included in the model training from publicly accessible online resources.
2. *GPT-4o ± REDCap*: The GPT-4o model plus material developed by the REDCap team at Vanderbilt University, including their own frequently asked questions (FAQs) and application help documentation. This comprised of approximately 74 000 words in a 0.8 megabyte help file.
3. *GPT-4o ± UM*: The GPT-4o model plus our locally developed UM REDCap support resources including FAQs and answers, our technology services manual, release guides, detailed work instructions, and boiler plate answers for commonly encountered issues. These resources were comprised of approximately 500 000 words in 82 megabytes of files that also contained screenshots and other images. Note that images (including embedded images within files such as PDFs, or text within images) were not interpreted by the models at the time of these experiments.
4. *GPT-4o ± REDCap ± UM*: The GPT-4o model plus a combination of the Vanderbilt-developed and UM locally developed documentation.

### LLM prompts

Each of the 4 LLM environments was tested using all 90 support tickets selected for this study. For each ticket the LLMs were prompted 3 times, corresponding to 3 distinct tasks: (1) assigning a complexity to the ticket (low, moderate, or high); (2) assigning the ticket to 1 of 11 support categories; and (3) drafting an e-mail response to the user based on the issue described in the ticket. The specific wording and structure of these tasks provided to the LLMs are detailed in Table 2. Ticket category assignments are not part of routine support team triage, but the team considered such categorization useful to have, especially if an AI tool could automate this step. For this study, the team developed these support categories collaboratively through group dialog and deliberation. While we recognize that alternative categorization schemes are possible, we selected this approach based on its relevance to our operational context and its practicality for the analysis at hand. Even though tickets could be multifaceted, both the humans and the LLMs assigned only one category to each ticket.

**Table 2:** Tasks used to construct prompts for the LLM experiments.

Topic	LLM task
Ticket complexity	We are going to classify REDCap support tickets by complexity, using these complexity definitions. Please respond with a single word answer. Low complexity—a straightforward question which is answered by the support team using standardized copy/paste responses or triggering of automated system replies, moderate complexity—a routine question that requires some degree of customized response or straightforward investigation by the support team, high complexity—an in-depth question that requires investigation and research by the support team and cannot be answered as a generalized reply: [Original Ticket]
Ticket category	We are going to classify REDCap support tickets by category, using these category definitions. Please respond with only the category name. **Account Management—**Tickets where users are unable to access REDCap for MICHR-supported account reasons (eg, New Account Setups, Suspended Account Reactivations, Sponsorship Renewals), **API Management—**Tickets where users are requesting support for the REDCap API or Mobile App (eg, Issue API Token, API Connectivity, API errors), **Field Functionality—**Tickets where users request support for field/form level behavior (eg, Calculations, Branching Logic, Validation, Field Types, Piping, etc.), **Login Issues—**Tickets where users are unable to access REDCap but should have an account and access (eg, Login/URL process issues, password resets, email verification), **Project Creation—**Tickets where users are requesting a new project be created (eg, Project Creation, Project Copy), **Project Management—**Tickets where users are requesting changes at the project level settings (eg, Design changes, Enable/Disable project-level features, event/instrument management, etc.), **Security—**Tickets regarding system or application security or Information Assurance (eg, risk assessments, technical security questions, compliance, and sensitive data removal), **Survey Design & Processes—**Tickets with questions about survey setup and design (eg, Survey Queue, Automated Survey Invitations, etc.), **System Technical—**Tickets regarding system or application technical issues (eg, outages/outage inquiries, system slowdowns, bugs), **Training Resources—**Tickets requesting training assistance or materials. **User Management—**Tickets where there are questions or issues with project-level User Rights (eg, DAGs, User Rights, Roles): [Original Ticket]
Response for user	We are going to answer REDCap application support questions to try and assist the user encountering this issue. Please respond as if you are providing support assistance for the following original ticket: [Original Ticket]

Note that the categories are shown in bold to make them easier to identify. They were not bolded in the prompt sent to the models. The original user-submitted help ticket was added to the prompt where it states “[Original Ticket]”.

Abbreviations: LLM, large language model; REDCap, Research Electronic Data Capture.

To streamline the prompt construction and ensure consistency, we employed a variable “piping” approach in which placeholders in square brackets (eg, [LLM task], [context], [Original Ticket]) were used within prompt templates. These placeholders were dynamically replaced with the relevant information for each experiment and ticket:

- [LLM task] represents the specific instruction or question the LLM was to answer, formulated according to the tasks shown in Table 2.
- [context] indicates supplemental material provided to the LLM. For Maizey RAG experiments, this consisted of the most relevant extracted passages from indexed documentation; for the GPT-4o Default environment, [context] was intentionally left blank, as no additional context was provided.
- [Original Ticket] denotes the user-submitted help ticket and serves as the core input for each prompt.

All 4 LLM environments were given the same overarching system prompt template:

Use the following pieces of context to answer the question at the end. If you don’t know the answer, just say that you don’t know, don’t try to make up an answer. [context] Question: [LLM task] Helpful Answer:

For each prompt sent to the LLM, the [context] and [LLM task] placeholders were programmatically replaced with the relevant extracted context and tasks description, as outlined above. This template-based approach enabled standardized and reproducible experimentation. For all prompts sent to the LLM the following recommended settings were used: (1) conversational; (2) do not return data sources; (3) temperature 1.0; (4) chunks: 4. The “conversational” options sets the overall tone for how the LLM responds and is a useful option for generating responses to help tickets. The “do not return data sources” option applies to RAG environments, where typically the LLM will provide supporting information from the original sources along with its answer. In this case, we wanted only the answer and not references to the source documentation. The “temperature” setting defines how much variability or creativity will be present in the model’s response. Lower temperatures are more predictable and conservative. A temperature of 1.0 is often a standard default value for many models. A chunk is a segment of text extracted from a source document, typically broken up by a RAG system to make information retrieval more efficient. We selected chunks = 4 (a common recommended default) to provide a balanced amount of relevant material for generating comprehensive answers, while maintaining both efficiency and conciseness in the responses.

### Human judgments

Our REDCap-trained staff performed judgments related to the tickets themselves as well as on the tasks completed by the AI.

The 3 REDCap staff individually reviewed the 90 tickets and grouped each ticket into low, moderate, or high complexity to serve as an official gold standard for complexity. A final gold-standard complexity score was then assigned to each ticket based on the majority consensus of the 3 reviewers. Interrater agreement was also calculated using the Fleiss’ kappa statistic with the “irr” package (version 0.84.1)^11^ in R 4.5.1. The human gold standard was then compared to each of the LLM environments using Cohen’s kappa statistics, also with the “irr” package.

For the support ticket categorization, the 3-member REDCap team collectively reviewed and discussed each ticket and determined a consensus gold-standard category. These categories are described in Table 2.

Finally, the simulated e-mail responses for users generated by the 4 LLM environment were reviewed by one individual (A.C.) and categorized as follows:

1. *Substantially or fully correct*: Artificial intelligence provided a response that contained no or only minor errors in instructions or tone/audience. Information provided would be helpful to user who submitted the ticket.
2. *Partially correct*: Artificial intelligence provided a response that had significant elements correct but also contained significant errors such as:
  - Was inconsistent in the audience (confused MICHR Support and the end user) and provided instructions one group or audience would not be able to complete.
  - Missed steps or checks that are important in the process but not necessarily critical.
  - Provided additional steps or instruction that were not helpful but also not harmful.
3. *Wrong*: Artificial intelligence was unable to provide an answer or the answer provided was completely wrong or included instructions that were harmful or counterproductive.

## Results

### Complexity ratings

The 3 human annotators had full agreement for complexity on 50 tickets, and agreed with a two-thirds majority on the remaining 40 tickets. There were no tickets in which all 3 annotators disagreed on complexity. The final gold-standard ratings based on a majority consensus included 55 low-, 31 moderate-, and 4 high-complexity tickets. The interrater agreement using the Fleiss’ kappa statistic was 0.43, which can be interpreted as moderate agreement.^12^

The AI judgments were then compared to the human gold-standard complexity scores to determine if the AI matched the gold-standard complexity, overestimated the complexity compared to gold standard, underestimated the complexity, or did not answer. The results are presented in Table 3. Overall, the 3 Maizey environments overestimated the complexity nearly 1.5 times as often as the Default AI system, but they underestimated the complexity less often than the Default system. The Default system had the highest number of exact matches on complexity based on the gold standard.

**Table 3:** Comparisons of each LLM environment to the gold-standard human judgments for ticket complexity.

	GPT-4o Default	GPT-4o + REDCap	GPT-4o + UM	GPT-4o + REDCap + UM
AI matched the gold-standard complexity	52 (58%)	40 (44%)	37 (41%)	34 (38%)
AI overestimated the complexity	23 (31%)	40 (44%)	44 (49%)	45 (50%)
AI underestimated the complexity	15 (17%)	9 (10%)	8 (9%)	9 (10%)
AI did not answer	0 (0%)	1^a^ (1%)	1^b^ (1%)	1^b^ (1%)
Cohen’s kappa	0.23	0.11	0.08	0.03

The first 3 rows report the number of tickets the LLM matched, overestimated, or underestimated the complexity. The bottom row is the agreement between the human and LLM judgments, which can be interpreted as “fair” for the default environment and “slight” for the other 3 environments.

a Human-judged complexity rating was low for the ticket that AI would not answer.

b Human-judged complexity rating was moderate for the ticket that AI would not answer.

Due to potential concerns about the validity of the sampling approach where ticket complexity was initially estimated and scored based on the total number of ticket updates and the total time required to resolve a ticket, we ran a Spearman rank correlation to assess whether higher estimated complexity scores (ranging from 2 = low to 7 = high) were associated with higher human-assigned categories (“low,” “moderate,” “high”). Our results showed a statistically significant, moderately positive relationship (rho = 0.31, *P* = .0025), indicating that as our estimated complexity scores increased, the likelihood of a higher human-assigned category also increased. This demonstrates a meaningful correspondence between the numerical estimates based on time and updates and human judgment.

### Ticket support category

**Figure 1:**
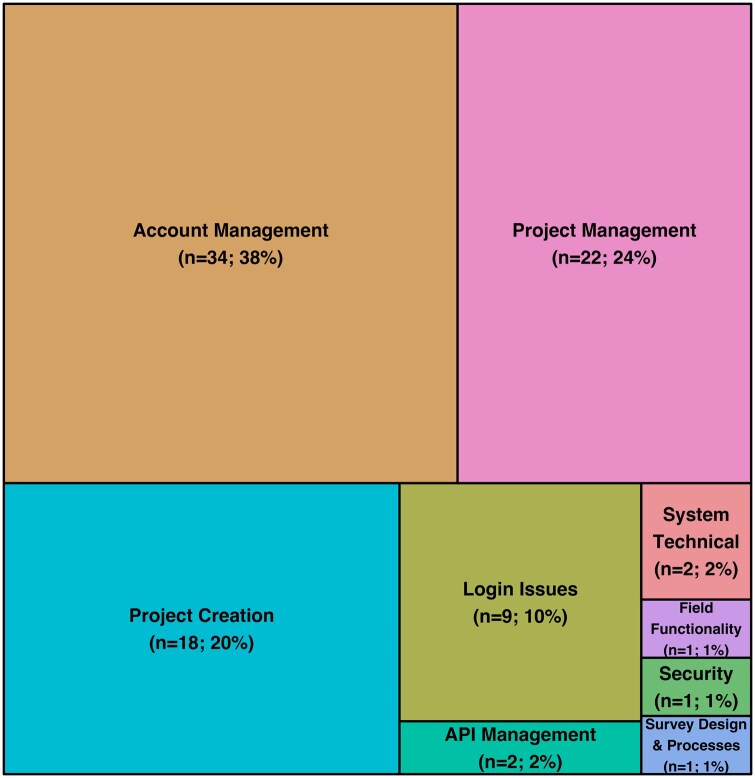
Treemap^13^ diagram showing the human categorizations of the 90 REDCap tickets used in this study. Two of the predetermined categories (training resources and user management) were not represented in the sample of tickets and are not shown in the plot. Abbreviation: REDCap, Research Electronic Data Capture.

The human reviewers categorized the 90 tickets into 1 of 11 categories, which are shown as a tree plot in Figure 1.

**Figure 2:**
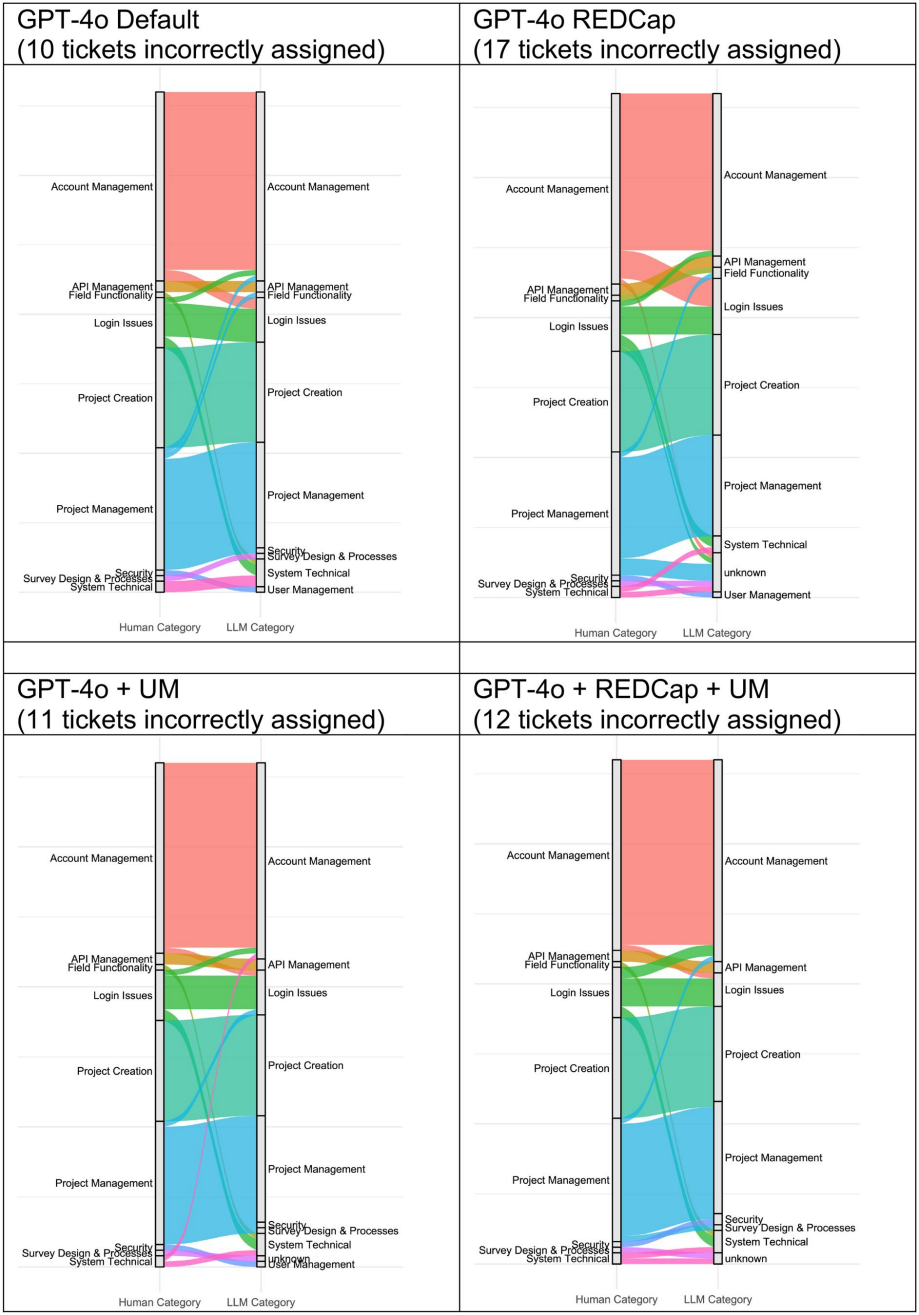
Alluvial plots^14^^,^^15^ showing the misclassification of tickets from the gold-standard human-generated categories (left side) to the LLM-generated categories (right side). To interpret these plots, ticket assignments that changed categories moving from left to right are those that were misclassified by the LLM, and the category on the right shows the specific category to which the ticket was classified. Tickets that have the same categories on both the left and the right side are those in which there was agreement between the humans and the LLM. Abbreviation: LLM, large language model.

When assigning tickets to 1 of the 11 categories, the LLMs performed reasonably well. The “GPT-4o Default” instance correctly assigned 80 (89%) of the tickets to the correct category, whereas the “GPT-4o + REDCap” instance correctly assigned 73 (81%), the “GPT-4o + UM” instance correctly assigned 79 (88%), and the “GPT-4o + REDCap + UM” instance correctly assigned 78 (87%). Ticket assignments to the incorrect category were variable and are shown in Figure 2. Cohen’s kappa for interrater agreement comparing the human judgment to each LLM environment was “almost perfect” for all environments: 0.85 for “GPT-4o Default,” 0.84 for “GPT-4o + REDCap,” 0.85 for “GPT-4o + UM,” and 0.85 for “GPT-4o + REDCap + UM.”

### LLM-generated responses

**Figure 3:**
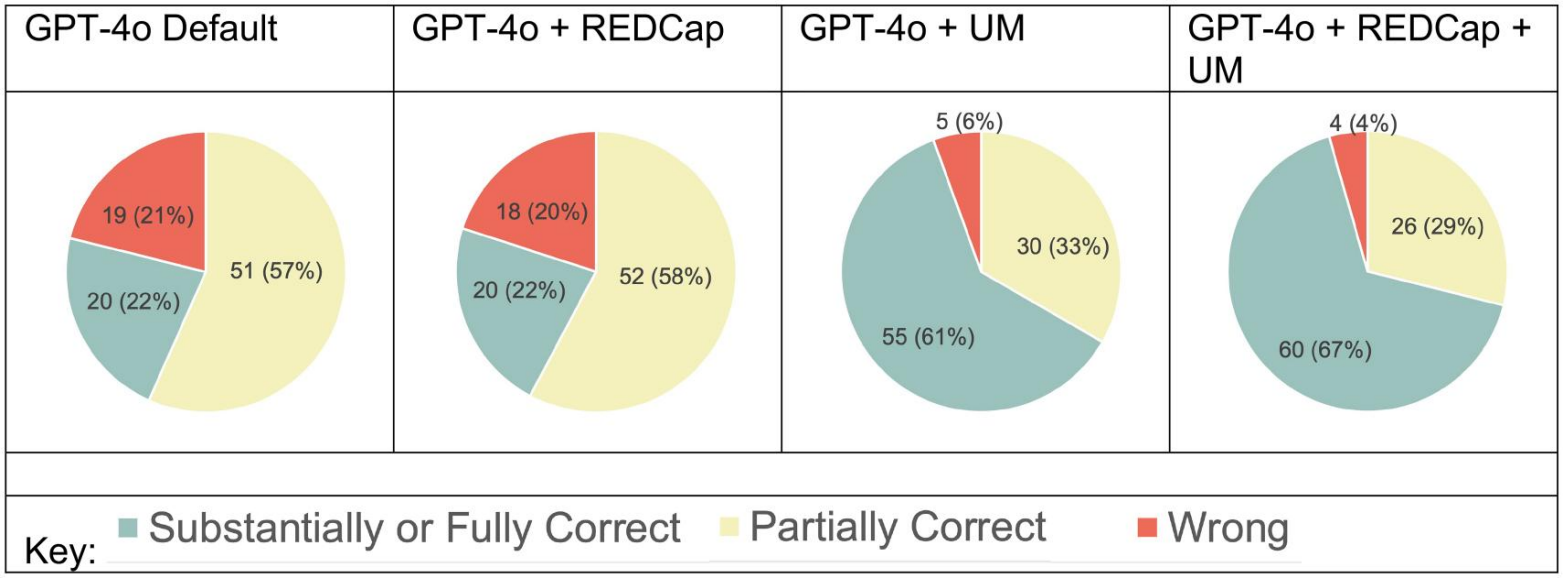
Accuracy ratings of ticket responses generated by the LLMs, as judged by a human reviewer. Abbreviation: LLM, large language model.

Responses (as messages to users) to the tickets generated by the LLMs were categorized based on level of correctness. The overall levels of correct, partially correct, and incorrect responses are shown in Figure 3. Details about the percentage of fully correct responses as a function of ticket complexity can be found in Table 4.

**Table 4:** Number (%) of tickets answered substantially or fully correct per category of ticket complexity for each of the LLMs tested.

Ticket complexity (as judged by humans)	Total tickets	GPT-4o Default	GPT-4o + REDCap	GPT-4o + UM	GPT-4o + REDCap + UM
Low	55	9 (16%)	9 (16%)	33 (60%)	37 (67%)
Moderate	31	10 (33%)	9 (29%)	21 (68%)	21 (68%)
High	4	1 (25%)	2 (50%)	1 (25%)	2 (50%)

The overall top scores were achieved by the GPT-4o + REDCap + UM where about two-thirds of the responses were substantially or fully correct for the low and moderate complexity ticket categories.

## Discussion

The results of our work suggest that LLMs may one day be able to assist with REDCap support tasks. However, like many studies involving LLMs, the results are somewhat mixed.^16^ Large language models were able to demonstrate the capacity to automate several REDCap support ticket tasks—complexity assessment, categorization, and drafting responses—with a range of accuracies depending on the configuration. The most context-enriched RAG LLM environment (GPT-4o + REDCap + UM) achieved the highest portion of substantially or fully correct responses for low and moderate complexity tickets (67% and 68%, respectively). But that still leaves about one-third of the responses with mistakes. High-complexity tickets remained challenging across all models, with only 25%-50% rated as correct, although this result needs to be interpreted in the context of only 4 tickets meeting the high-complexity criteria in the sample, limiting the robustness of these findings. Other research has also shown LLMs backed with RAG can provide reasonable customer support responses,^17^ but that that LLM-generated responses may need additional review for complex customer questions.^18^

With regard to ticket complexity assessment, the GPT-4o Default environment show the most exact matches (58%). The context-enriched (Maizey) RAG environments tended to overestimate complexity more than the Default model, but they underestimated complexity about half as much as the default model. In terms of supporting time management for the team, it may be more harmful to underestimate complexity rather than overestimating. All the LLM environments demonstrated limitations in matching the human consensus, highlighting the nuanced judgment needed for accurate ticket triage, which remains an active area of research.^19^ Accurate category assignment is important because it ensures that tickets are triaged directly to the correct staff member, facilitating faster resolution of user problems and more efficient use of support resources.

All LLMs performed relatively well (81%-89% accuracy) performing the ticket category assignments, with the Default system the top performer. This could be because these categories were developed for the study and are not found in the documentation used to augment the LLM environments. However, it is also worthwhile to note that the additional documentation in the context-enriched environments resulted in worse performance. Other research has found variable accuracy when categorizing technical support tickets.^19^

The finding that adding more REDCap information to the RAG environment actually decreased accuracy in category assignment has important real-world implications. Institutions implementing AI chatbots for REDCap support should consider that providing extra local documentation does not always improve performance and in some cases may lead to less accurate triage and category assignments. This could be because the LLM is distracted by irrelevant details or has difficulty synthesizing large amounts of uneven documentation, particularly when the support taxonomy is not represented in the augmented materials. For administrators, this means that utilizing an “off-the-shelf” AI platform such as ChatGPT, without additional context enrichment, may be reasonable for tasks like triage or category assignment, provided that the core REDCap knowledge is captured within the base model. In contrast, when the task is to draft substantive responses to user questions, our results suggest that enriching the context with relevant REDCap and local institutional information yields the best performance. Therefore, the level of documentation augmentation should be intentionally determined based on the support task being automated, and not assumed to be universally beneficial.

The substantial number of straightforward, low and moderate complexity tickets submitted by our user base suggests that LLMs could offload a meaningful proportion of the REDCap support teams’ workload if integrated carefully. Utilizing LLMs for initial triage and draft response generation could reduce support staff response times, freeing them to focus on higher complexity issues requiring specialized expertise.^20^ The tendency for LLMs to overestimate complexity could be seen a benefit, reducing the risk of undertriaging challenging tickets.

This study has several strengths. First, it draws from the real-world and diverse set of support tickets collected at a large research institution with a robust REDCap user community. Second, we utilized multiple LLM environments that were systematically compared to standardized tasks. Our study also has some notable limitations. Our sample of 90 tickets, although stratified by estimated complexity, may not fully capture the variety of issues encountered by our user base. Our complexity estimates were based on time to resolution and number of ticket updates, which are crude, and likely imperfect, measures of how complex a ticket might be to resolve. Further, the complexity estimates themselves yielded only 4 complex tickets for testing, giving us fewer opportunities to test these LLM environments in the most challenging cases. Only one human reviewer graded the AI-generated response for correctness. Future work should include multiple raters for all comparisons. Last, it should be noted that the LLMs themselves demonstrated some limitations not reported in the results. The LLMs were instructed to answer with simple responses, such as one word for complexity or only the name of the support category. However, they sometimes answered with lengthy responses that contained extraneous details. These responses were manually cleaned before conducting the analysis, but they could have an impact if the LLMs were incorporated into other workflow systems relying on consistent output.

Interestingly, our results also reveal that the complexity scores based on ticket updates and resolution time were often discordant with human ratings of ticket complexity (eg, only 4 tickets were classified as high complexity by human review, contrasting with 30 initially sampled from the 2003 identified by the proxy metric in Table 1). While this discordance represents a limitation in using automated or proxy measures to estimate complexity, it simultaneously highlights a future opportunity. From a process management perspective, cases where AI-generated complexity scores differ substantially from the real-world updates and resolution time could flag tickets for workflow review. Harnessing these discordant cases could help managers iteratively refine triage criteria, streamline support workflows, and improve team efficiency.

## Conclusion

Overall, the use of LLMs for drafting responses to low and moderate complexity tickets appears feasible, with substantial human labor savings possible after a manual review for correctness. Ongoing human oversight remains critical, particularly for nonroutine or high-complexity queries and for the continuous refinement of AI-augmented support tools. Additional prompt engineering, as well as expansion of local documentation for the RAG system could yield further improvements. Future work should explore the user’s (researcher’s) satisfaction with AI-generated responses and assess whether more efficient support ultimately leads to enhanced research productivity.

Additional future work could explore the value of using an ensemble of LLMs and having these models independently process and then cross-check ticket assignments and draft responses. Approaches such as comparing concordance in category classification or having one LLM assess the output of another may further reduce the need for time-intensive human reviews, while increasing triage accuracy and reviewer confidence. This strategy has shown promise in other domains, especially when rapid, low-cost LLM processing is available compared to more costly and time-intensive human adjudication.

## Data Availability

The judgments from the reviewers, as well as the judgments made by the LLMs can be provided upon request. The user-submitted tickets and the email responses generated by the LLMs are not deidentified and can be made available through a data use agreement with the University of Michigan.
